# Curettage or Resection? A Review on the Surgical Treatment of Low-Grade Chondrosarcomas

**DOI:** 10.7759/cureus.39637

**Published:** 2023-05-29

**Authors:** Filipe Castelo, Afonso Faria, Hugo Miranda, Vânia Oliveira, Pedro Cardoso

**Affiliations:** 1 Orthopaedics and Trauma, Centro Hospitalar Universitário de Santo António, Porto, PRT; 2 Orthopaedics and Trauma, Centro Hospitalar Universitário Cova da Beira, Covilhã, PRT; 3 Oncology, Centro Hospitalar Universitário de Santo António, Porto, PRT; 4 Oncology, Unit for Multidisciplinary Research in Biomedicine, ICBAS-UP, Porto, PRT; 5 Orthopaedics, Centro Hospitalar Universitário de Santo António, Porto, PRT

**Keywords:** resection, curettage, surgical treatment, atypical cartilaginous tumor, low-grade chondrosarcoma

## Abstract

Introduction

Low-grade chondrosarcomas (LG-CS), including atypical cartilaginous tumors (ACT), are locally aggressive lesions. The focus of the discussion sits on the differential diagnosis between benign lesions or aggressive cartilaginous tumors and on their treatment: intralesional curettage or wide resection. This study presents the results obtained in the surgical treatment of 21 cases of LG-CS.

Methods

This retrospective study includes 21 consecutive patients from a single center with LG-CS who underwent surgery from 2013 to 2021. Fourteen were located in the appendicular skeleton, and seven in the axial (shoulder blade, spine, or pelvis). Mortality rate, recurrence, metastatic disease, overall survival, recurrence-free survival, and metastatic disease-free survival were analyzed for each type of procedure and each disease location. Operative complications and residual tumors were also recorded in cases where resection was performed. Survival was calculated using the Kaplan-Meier method.

Results

Thirteen patients underwent intralesional curettage (11 appendicular and 2 axial lesions), and eight underwent wide resection (5 axial and 3 appendicular). There were six recurrences during the follow-up, 43% of the axial lesions recurred, rising to 100% in axial curetted ones. Appendicular LG-CS recurred in 21% of cases, and only 18% of curetted appendicular lesions were not eradicated. The overall survival for the entire follow-up is 90.5%, and the 5-year survival rate is 83% (12 patients have adequate follow-up). Recurrence-free and metastasis-free survival were higher in resection cases, with 75% and 87.5%, vs. curettage 69.2% and 76.9%, respectively. In 9% of cases, the preoperative biopsy was inconsistent with the pathology of the surgical specimen.

Discussion

LG-CS and ACT are described as having high survival and low potential for metastatic disease. For this reason, these lesions are subject to a change in treatment philosophy to reflect these characteristics. Intra-lesional curettage is advocated as a less invasive technique for eradicating atypical cartilage tumors and has fewer and less severe complications, which was in accordance with our findings. Diagnosis, however, is challenging; misgrading is frequent and should be considered. Because of this risk of under-treating higher-grade lesions, some authors still defend wide-resection as the treatment of choice. We observed a trend towards longer survival, less recurrence, and metastatic disease with wide resection. Metastatic disease was higher than expected, present in 19% of cases, and always associated with local recurrence.

Conclusion

LG-CS is still a diagnostic and treatment challenge; patient selection is fundamental. Overall survival is high, independent of treatment choice or lesion location. We found a higher rate of metastatic disease than described in the literature; this, coupled with a misgrading rate of 9%, reflects the difficulty of preoperative diagnosis and the risk of treating high-grade chondrosarcomas as a low-grade lesion. More studies should be carried out with larger samples to obtain statistically robust results.

## Introduction

Classical chondrosarcomas (CS) are malignant cartilaginous lesions with significant heterogeneity. These lesions can have different degrees of aggressiveness according to Evans et al.: grade 1, low grade (LG-CS) or atypical cartilaginous tumor (ACT), grade 2 (G2), grade 3 (G3) and its dedifferentiated form. [1] CS is the third most frequent bone tumor and the leading cause of adult bone sarcoma. They occur mostly during the fourth and fifth decades of life. The most affected regions are the pelvis and proximal femur [1].

According to the World Health Organization (WHO), LG-CS refers to lesions in the axial skeleton (pelvis, spine, scapula, and skull), and ACT refers exclusively to lesions in long bones. In this report, the term LG-CS will be used except when referring specifically to long bone lesions. The WHO definition is based on the less aggressive nature of these lesions (clinically, pathology, and imaging characteristics) when present in the long bones. [1] These lesions are also divided between primary lesions, "de novo" CS, or secondary to a previously existing benign lesion such as an enchondroma. The location where the CS forms define whether it is a central (intraosseous) or peripheral (exostotic) lesion. [1] Higher-grade CS are usually clearly malignant by their histological appearance and are easily identified; however, LG-CS are hardly distinguishable from benign chondral neoplasms. The histological features are hyaline cartilage matrix, relative hypercellularity, and trabecular permeation. [2,3] Clinical and imaging valuable signs include pain and signs of aggressiveness, such as endosteal scalloping, cortical thinning, lesion growth, calcification changes, and pathological fractures [4].

LG-CS are mildly aggressive lesions with a favorable long-term prognosis; recurrence and metastasis are infrequent, and survival is long, especially in long bones. For this reason, less invasive treatments have been gaining ground to decrease the clinical and social impact on these patients [5,6].

When an LG-CS is suspected, the studies should include clinical and imaging factors. [2] According to some authors, a histological study by biopsy should be reserved for cases where doubt remains as to the aggressiveness and staging of the lesion. [7] Due to the heterogeneity of LG-CS, biopsy should be carefully planned since different degrees of aggressiveness or even regions of benign histological appearance may coexist in the same lesion [8,9].

CS are chemo- and radio-resistant. Thus, curative treatment is surgical. Wide resection is generally accepted as the gold standard when discussing CS G2, G3, and LG-CS of axial location. As for ACT, there is still no consensus among the scientific community regarding the best treatment option [10,11].

ACTs treatment may range from surveillance for clinical (onset of pain) and imaging changes to intralesional surgery with aggressive curettage with or without adjuvants such as phenol, drilling or cryoablation, or wide resection [2]. The advantages of less invasive techniques are lower complication rates and less postoperative functional deficit. Proponents of curettage claim that the low aggressiveness of ACTs, rare metastatic disease and recurrence, and long survival are reasons these lesions can be treated with less invasive procedures [12]. On the other hand, some surgeons advocate wide resection even for ACTs because the misgrading rate is high due to difficult imaging and histological interpretation of these lesions and may lead to under-treatment of true high-grade tumors [11].

The present retrospective study aims to address an adequate surgical treatment for LG-CS and analyze clinical results regarding local recurrence, metastatic disease development, mortality, and operative complications for each procedure type and location.

## Materials and methods

Patient selection

Twenty-three consecutive cases of LG-CS were collected retrospectively from our center's database. The data was collected from the multidisciplinary discussion meeting for the musculoskeletal tumors registry. The collected data encompasses the years from January 2013 to December 2021. Data was reviewed based on clinical records from the multidisciplinary discussion meeting, MRI and CT imaging reports, pathology reports, and surgery reports.

Inclusion criteria

We included all patients with a radiological and MRI or CT study compatible with LG-CS and a concordant pre- and postoperative histological study. Two cases were excluded because of diagnostic discordance between the preoperative study and the postoperative histological study. In both of these cases, the postoperative diagnosis was a high-grade chondrosarcoma. Equating to a preoperative misgrading rate of 9%. Of the 21 patients, 14 had ACT in long bones and 7 LG-CS in the axial skeleton. All patients were treated surgically: 13 intralesional curettages and 8 wide resections.

Statistical analysis

The mortality rate, recurrence, metastatic disease, overall survival, recurrence-free survival, and metastatic disease-free survival were analyzed for each type of procedure (curettage or resection) and each disease location (axial or long bones). Operative complications and residual tumor (R) were also recorded in cases where resection was performed. Survival was calculated using the Kaplan-Meier method. The software used was SPSS® Statistics 26.

## Results

The mean age of the population was 44 years (23-68), with 12 males (57%) and 9 females (43%). The mean follow-up was 54 months (15 - 121 months) (Table 1). Twenty-one patients with a diagnosis of LG-CS/ACT underwent surgical treatment. Thirteen aggressive curettage with adjuvants (drilling, phenolization, and polymethylmethacrylate (PMMA) space-filling) and 8 wide resections were performed (Table 2). Five resections were achieved with a residual tumor score of R0; one was an R1 (iliac bone), one was R2 (dorsal vertebra), and another could not be assessed (proximal femur). Of the 13 aggressive curettages, 11 were performed on long bone lesions (4 proximal femur, 2 distal femur, 1 distal radius, 1 proximal tibia, 2 proximal humerus, and 1 metacarpal) and 2 on axial lesions (2 acetabulum). Of the 8 wide resections performed, 3 were long bone lesions (2 proximal femur, 1 humerus), and 5 were axial lesions (2 dorsal spine, 1 scapula, 2 iliac bone) (Table 3). Patient data is provided in detail in Table 4. 

**Table 1:** Population age and sex

21 patients	
Mean age	44 (23-68)
Male	12 (57%)
Female	9 (43%)

**Table 2:** Procedure type by location

Procedure\Location	Axial	Appendicular	Total
Curettage	2	11	13
Resection	5	3	8
Total	7	14	21

**Table 3:** Primary disease anatomic location

Location	Total
Spine	2
Scapula	1
Proximal humerus	2
Diaphyseal humerus	1
Distal Radius	1
Hand	1
Pelvic	4
Proximal femur	6
Distal femur	2
Proximal tibia	1

**Table 4:** Population description R: Residual Tumor; FU: Follow-up; THR: Total Hip Replacement; L. Recurrence: Local Recurrence; LG: Low-Grade; G2: grade 2; G3: Grade 3; NA: Not applicable; NED – No Evidence of Disease; AWD – Alive with Disease; DOD – Death from Disease Progression; DOC – Death from Other Causes; PIPJ – Proximal Interphalangeal Joint

Patient	Age	Location	Procedure	R	FU	Complications	Local Recurrence	Time to recurrence	Recurrence grade	Metastatic disease	Status
1	26	Proximal fémur	Resection	R0	121	Trendlenburg gait	No	NA	NA	No	NED
2	45	Dorsal vertebra	Resection	R0	107	No	Yes	41	G2	Yes	AWD
3	23	Proximal humeurs	Curettage	NA	99	No	No	NA	NA	No	NED
4	39	Dorsal vertebra	Resection	R2	86	Seroma	No	NA	NA	No	NED
5	45	Acetabulum	Curettage	NA	83	THR instabiity	Yes	32	G2	Yes	AWD
6	59	Proximal humerus	Curettage	NA	81	Supraspinatus tear	No	NA	NA	No	NED
7	31	Proximal fémur	Resection	RX	77	Trochanteric pain	No	NA	NA	No	NED
8	54	Proximal fémur	Curettage	NA	56	No	Yes	35	Dedifferentiated	Yes	AWD
9	55	Proximal femur)	Curettage	NA	50	No	No	NA	NA	No	NED
10	29	Humerus	Resection	R0	47	Radial palsy (temporary)	Yes	22	LG	No	AWD
11	52	Proximal tíbia	Curettage	NA	47	No	No	NA	NA	No	DOC (stroke)
12	32	Iliac bone	Resection	R0	46	No	No	NA	NA	No	NED
13	45	Proximal fémur	Curettage	NA	38	No	Yes	18	G3	Yes	DOD
14	36	Distal femur	Curettage	NA	35	No	No	NA	NA	No	NED
15	50	Proximal fémur	Curettage	NA	28	No	No	NA	NA	No	NED
16	61	Distal femur	Curettage	NA	26	No	No	NA	NA	No	NED
17	47	Acetabulum	Curettage	R0	23	No	Yes	23	G2	No	AWD
18	68	Iliac bone	Resection	R1	22	Infection	No	NA	NA	No	NED
19	41	Scapula	Resection	R0	22	No	No	NA	NA	No	NED
20	34	Metacarpal	Curettage	NA	21	PIPJ rigidity	No	NA	NA	No	NED
21	60	Distal radius	Curettage	NA	15	No	No	NA	NA	No	NED

**Figure 1:**
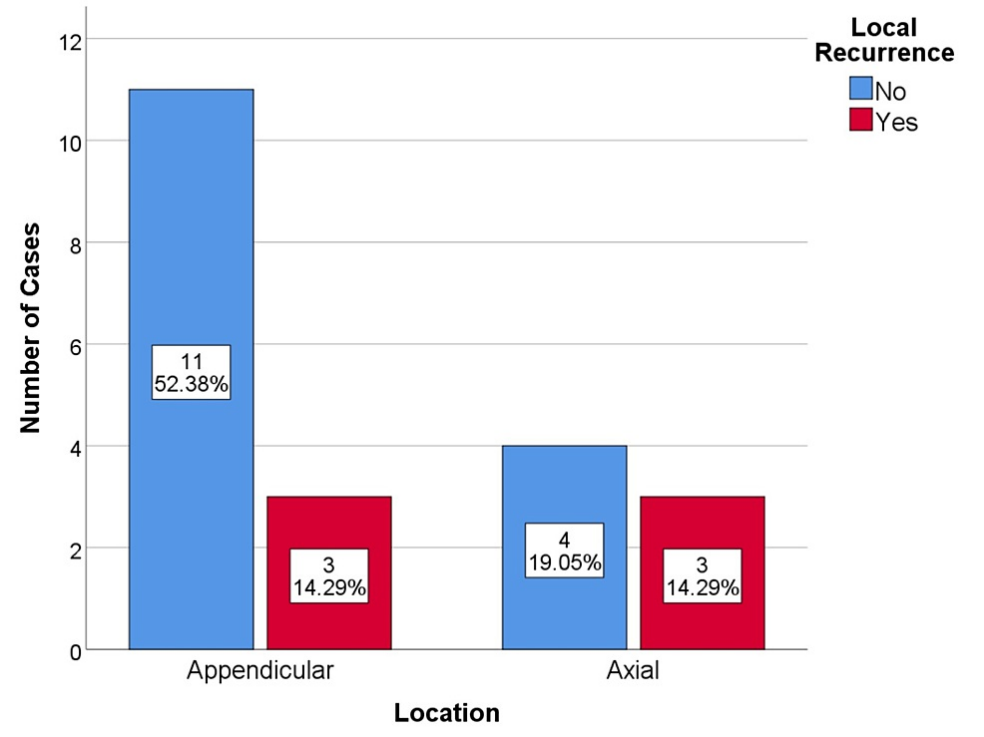
Local recurrence by tumor anatomic location, p=0.299

**Figure 2:**
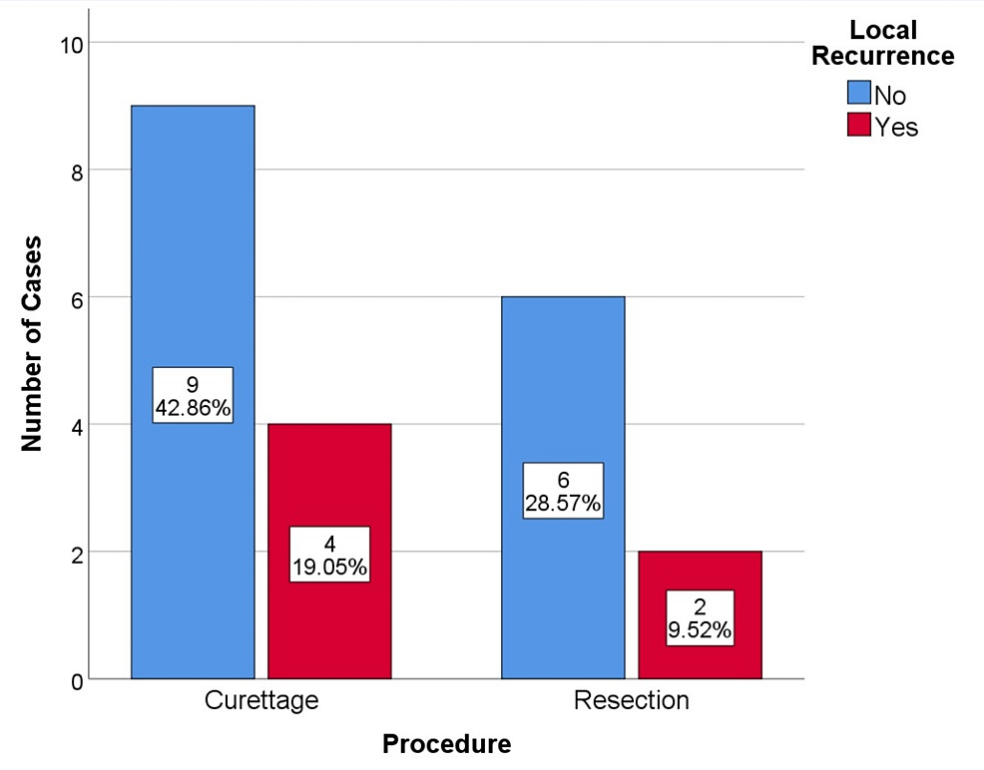
Local recurrence by procedure, p=0.509

A total of six local recurrences (29%), 3 in axial lesions (43%), and 3 in long bones (21%) were identified (Figure 1). In the two cases where curettage was performed on axial lesions, the local recurrence rate was 100% (both were acetabular lesions in which the procedure was aggressive curettage and total hip arthroplasty). On the other hand, the recurrence rate in the curetted peripheral lesions was much lower, at 18%. In the lesions that underwent wide resection, two recurrences occurred, which corresponds to a recurrence rate of 25%. The difference in recurrence rate is more evident when comparing curetted versus resected axial lesions: 100% vs. 20%, respectively (Figure 2). The residual tumor classification of R1 or R2 was not concurrent with a worse prognosis; there was no increase in recurrence, metastatic disease, or mortality rate in these cases. Data regarding recurrence, metastatic disease, and mortality is available in Tables 5-7.

**Table 5:** Local recurrence, metastatic disease and mortality by procedure in all anatomic locations 1 - p-Value refers to results from bivariate analysis comparison between curettage and resection groups for each variable, using chi-square or Fisher's exact tests.

All locations	Curettage	Resection	Total	p-value^1^
Local recurrence	4 (30.8%)	2 (25%)	6 (28.6%)	0.590
Metastatic disease	3 (23.1%)	1 (12.5%)	4 (19%)	0.502
Mortality	2 (15.4%)	0 (0%)	2 (9.5%)	0.505

**Table 6:** Local recurrence, metastatic disease and mortality by procedure in the axial skeleton 1 - p-Value refers to results from bivariate analysis comparison between curettage and resection groups for each variable, using chi-square or Fisher's exact tests.

Axial skeleton	Curettage	Resection	Total	p-value^1^
Local recurrence	2 (100%)	1 (20%)	3 (42.9%)	0.143
Metastatic disease	1 (50%)	1 (20%)	2 (28.6%)	0.524
Mortality	0 (0%)	0 (0%)	0 (0%)	-

**Table 7:** Local recurrence, metastatic disease and mortality by procedure in the appendicular skeleton 1 - p-Value refers to results from bivariate analysis comparison between curettage and resection groups for each variable, using chi-square or Fisher's exact tests.

Appendicular Skeleton	Curettage	Resection	Total	p-value^1^
Local recurrence	2 (66.7%)	1 (33.3%)	3 (21.4%)	0.547
Metastatic disease	2 (18.2%)	0 (0%)	2 (14.3%)	0.604
Mortality	2 (18.2%)	0 (0%)	2 (13.3%)	0.604

**Figure 3:**
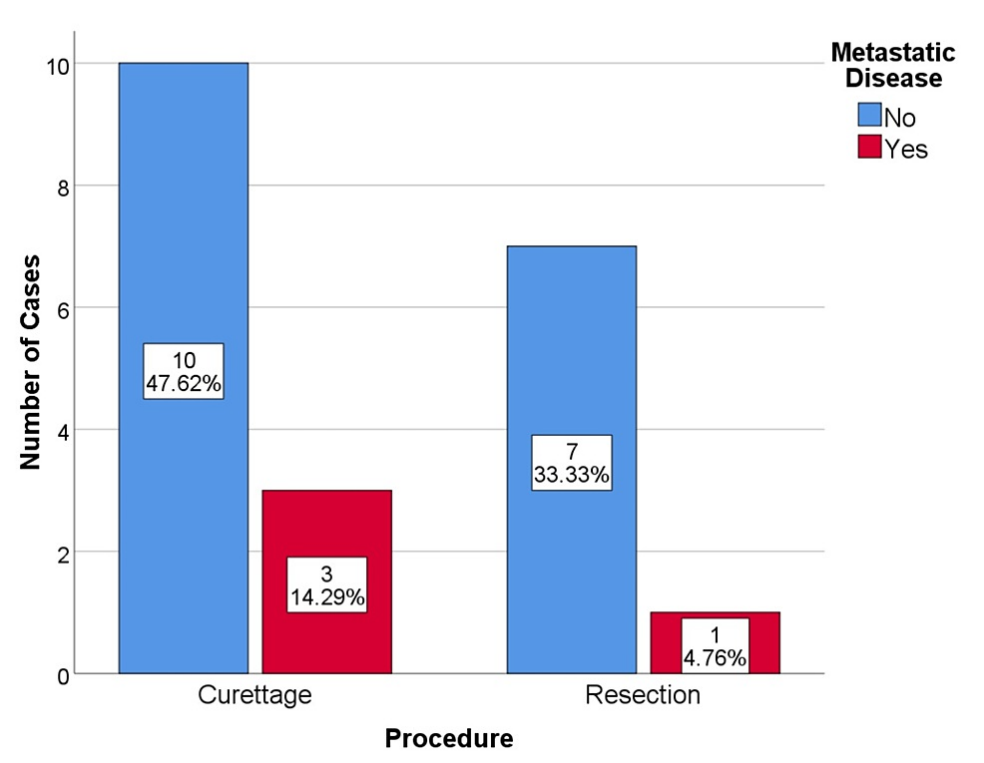
Metastatic disease by procedure, p=0.502

**Figure 4:**
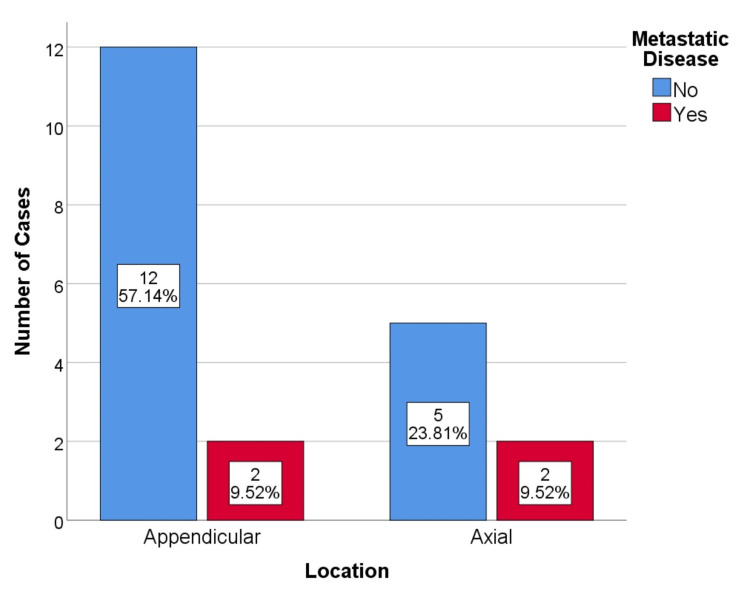
Metastatic disease by tumor anatomic location, p=0.407

No cases had metastatic disease during the initial study. During follow-up, four cases of metastatic disease were identified in patients with local recurrence. This represents a 19% metastatic disease rate. One case occurred in a resected dorsal spinal lesion, and the other three cases occurred in curettages (1 proximal femur, 1 humerus, and 1 acetabulum) (Figure 3, 4). All cases of metastatic disease coincided with disease upgrading. Two cases of G2, 1 case of G3 who died of disease progression, and 1 case of dedifferentiated chondrosarcoma. The metastatic vertebral chondrosarcoma was initially resected with free margins, R0.

**Figure 5:**
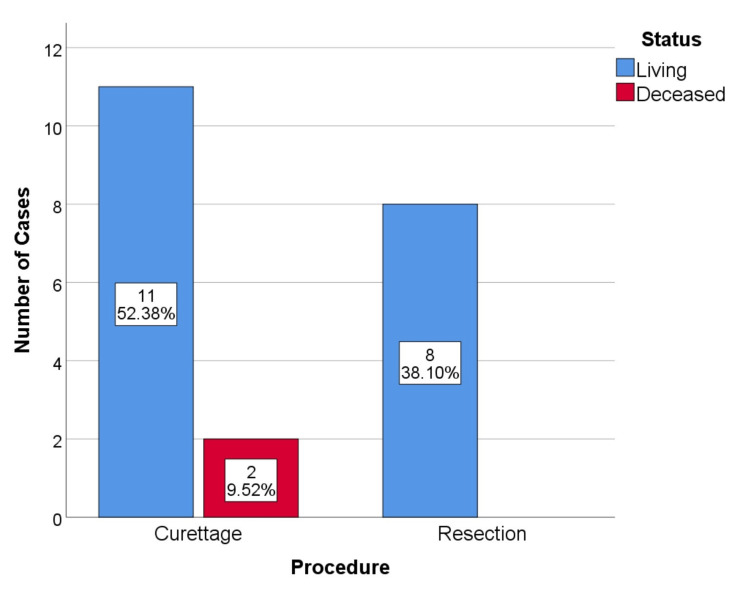
Mortality by procedure, p=0.505

**Figure 6:**
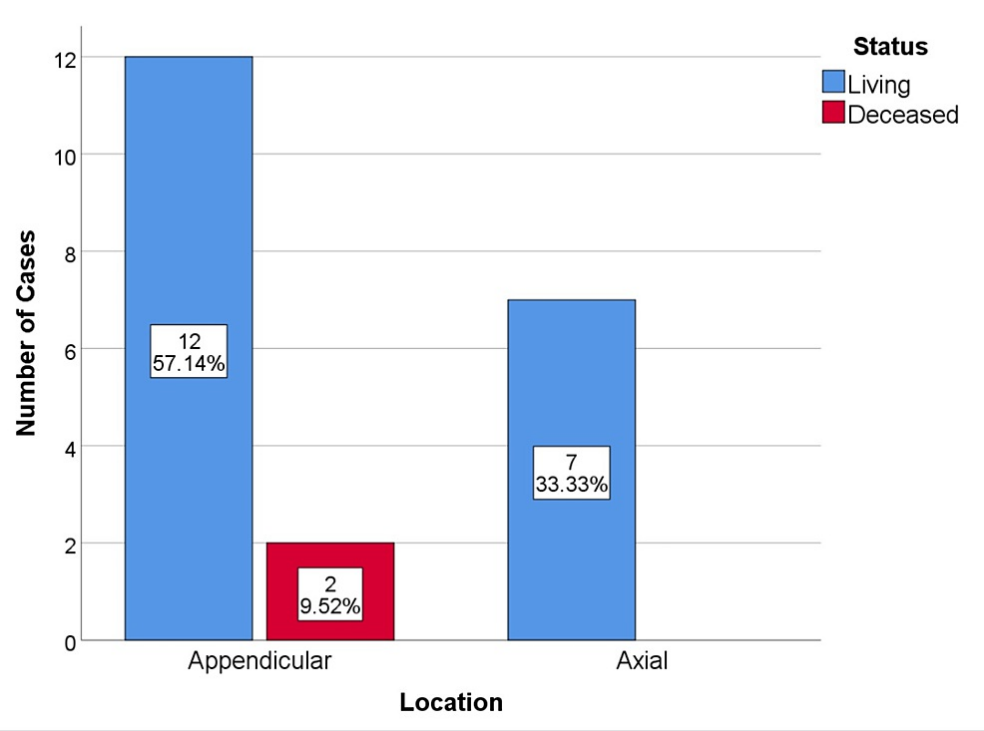
Mortality by tumor anatomic location, p=0.505

During follow-up, two deaths occurred within our population, one directly related to the disease, in a 45-year-old woman with an ACT of the proximal femur, who died 38 months after diagnosis due to disease recurrence with upgrading to a G3 CS with metastatic disease. The other death was due to a stroke in a 52-year-old man with an ACT of the proximal tibia submitted to curettage, with no evidence of recurrence and a follow-up of 47 months. If we exclude the latter case, the mortality rate directly related to the underlying disease is 5% (Figure 5, 6).

**Figure 7:**
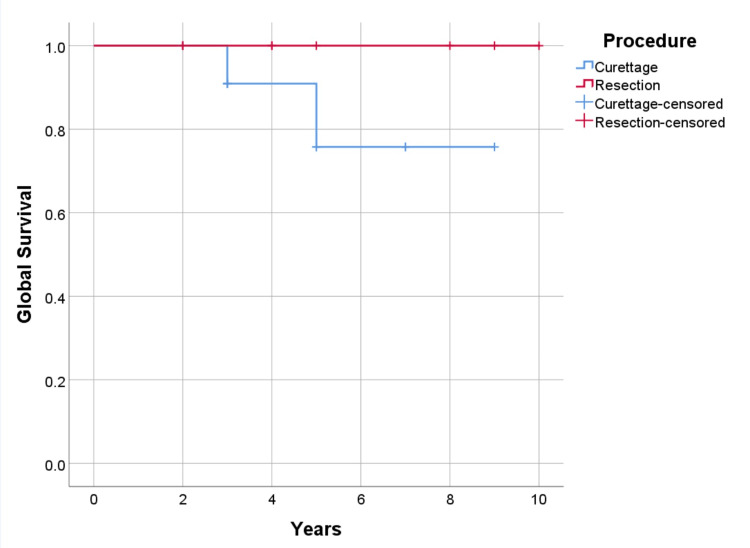
Global survival by procedure, p=0.271

**Figure 8:**
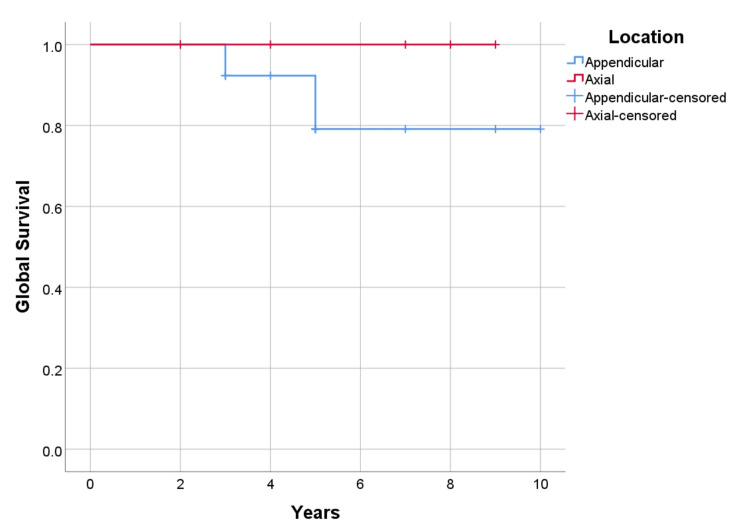
Global survival by anatomic location, p=0.391

The overall survival for the entire follow-up, calculated using the Kaplan-Meyer method, is 90.5% (100% for cases undergoing resection and 84.6% for curettage). The one-year survival was 100% (21 cases in follow-up), at 3-year survival was 94% (17 cases with follow-up), and five-year survival was 83% (12 cases with adequate follow-up) (p=0.271) (Figure 7, 8). Overall recurrence-free survival for the whole follow-up time was 71.4%. The overall recurrence-free one-year survival was 100%; at three years, 88% (follow-up 17 patients), and at five years, 69% (analyzing 13 patients) (p=0.656).

**Figure 9:**
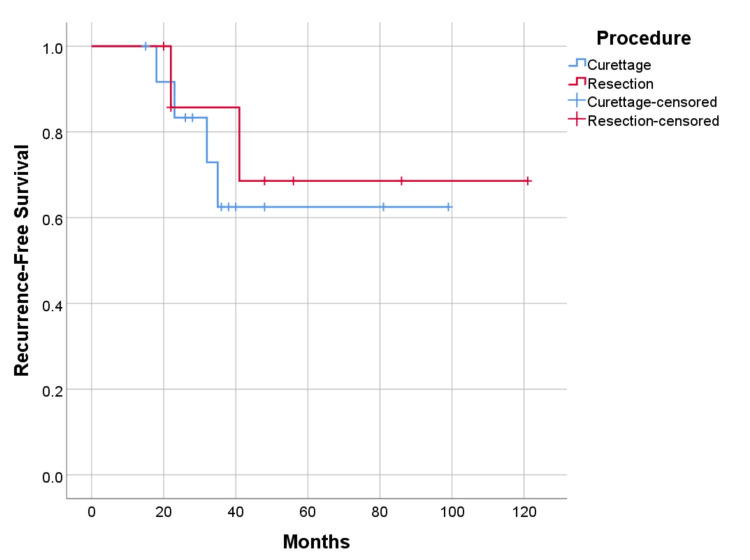
Recurrence-free survival by procedure, p=0.656

**Figure 10:**
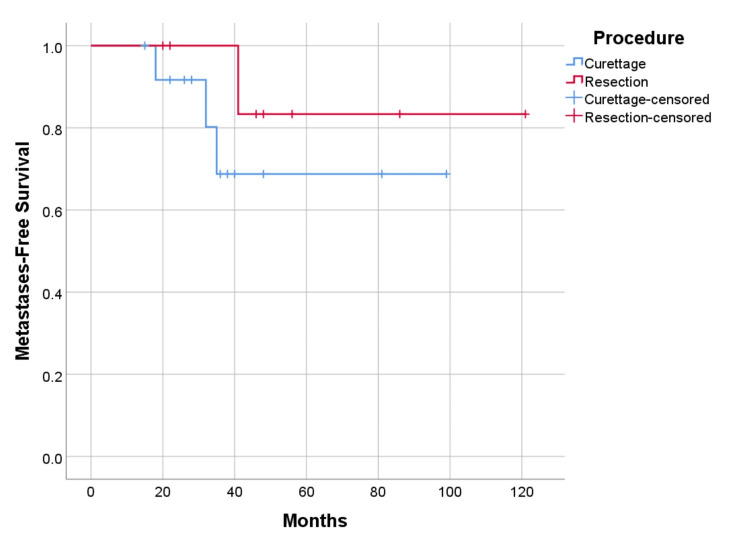
Metastases-free survival by procedure, p=0.328

**Figure 11:**
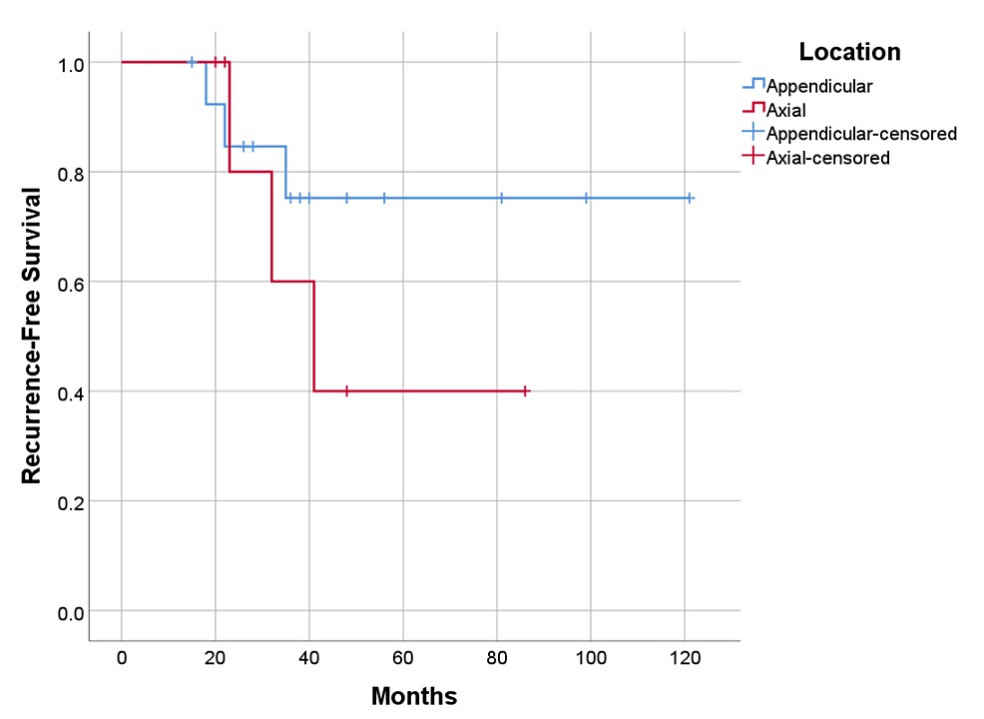
Recurrence-free survival by anatomic location, p=0.347

**Figure 12:**
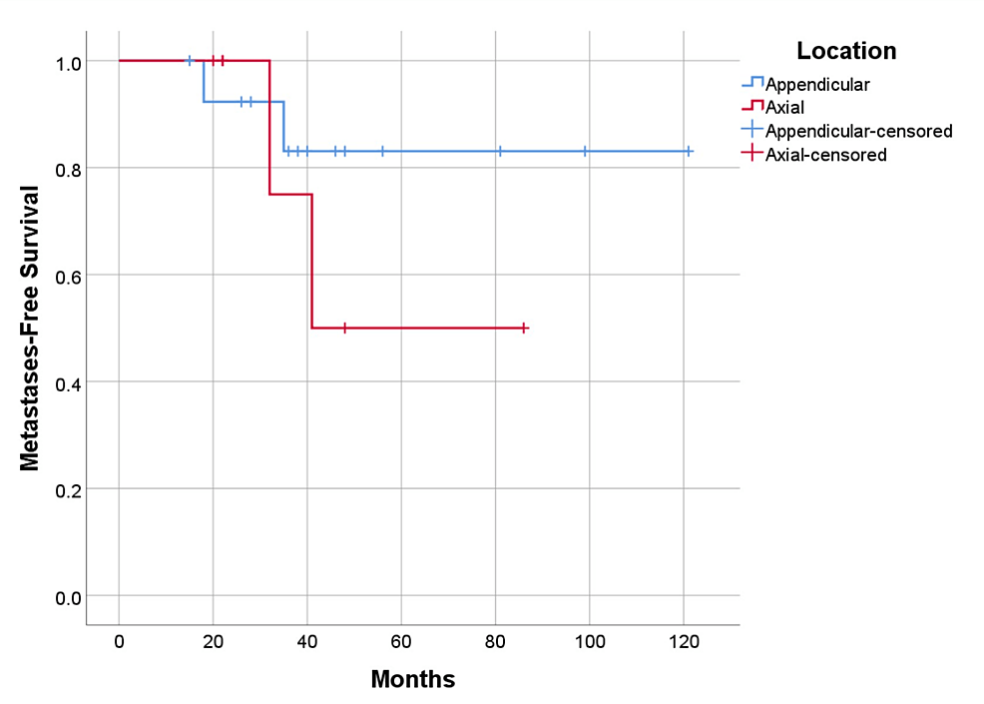
Metastases-free survival by anatomic location, p=0.380

Recurrence-free and metastasis-free survival were higher in cases undergoing resection with 75% (p=0.656) and 87.5% (p=0.328), versus curettage 69.2% (p=0.656), and 76.9% (p=0.328) respectively (Figure 9, 10). As for the location of the disease, survival is higher in cases with axial lesions than in appendicular ones, 100% vs. 85.7% (p=0.391). However, recurrence-free and metastasis-free survival are higher in lesions located in the appendicular skeleton than in the axial skeleton, respectively 78.6% vs. 57.1% (p=0.347) for recurrence, and 85.7% vs. 71.4% (p=0.380) for metastatic disease (Figure 11, 12). Global metastatic disease-free survival is 81% (p=0.380). Although there is a trend towards higher survival and metastasis-free survival for patients who underwent wide resection, we did not achieve statistical significance for any of these outcomes due to our sample size.

The two cases excluded for disagreeing preoperative (LG-CS) and postoperative (G2) studies correspond to a misgrading rate of 9%. Eight complications occurred during follow-up, 5 after resection (62.5%), and 3 after curettage (23%). Of these, only two cases required revision surgery, one for infection of hip arthroplasty with a mega-prosthesis (wide resection surgery) and the other for instability of primary hip arthroplasty (curettage with adjuvants of the acetabulum and primary hip arthroplasty). Of the remaining 6 that were not re-operated, three cases remained with sequelae (Trendlenburg gait, supraspinatus rupture, and digital stiffness), and three had spontaneous resolution (trochanteric bursitis, postoperative seroma, and radial nerve neuropraxia). If we subtract the cases with mild and self-resolving complications, we are left with 5 cases requiring re-intervention or having permanent sequelae. Two cases occurred in patients undergoing resection (25%), infection, and Trendlenburg gait. The remaining three complications occurred in curetted patients (13%), digital stiffness, supraspinatus tendon rupture, and hip arthroplasty instability.

## Discussion

The 5-year survival of LG-CS can be over 90%, and it rarely presents metastatic disease [6,13,14]. The current controversy lies in their correct diagnosis, grading, and treatment. To date, the generally accepted recommendations for treating LG-CS are wide resection in axial lesions and aggressive curettage or resection in the appendicular ACT [10,12,15-18].

Some authors defend a conservative or less invasive approach for ACTs based on radiological and magnetic resonance imaging findings alone. If appendicular lesions do not present imagiological findings associated with higher-grade CS, they should be treated with curettage or as "don't touch" lesions [19].

The overall survival for this disease is high whether patients undergo curettage or wide resection in both axial and appendicular lesions. The result obtained in our series is similar in this respect to that described in the literature [6,18]. However, the average follow-up of 54 months is short. Especially since the decrease in survival is felt after 10 years of follow-up [20]. The local recurrence rate of 28.6% was higher than that described in the literature, between 0 and 26% [1,18,21,22].

Current recommendations for treating LG-CS are curing appendicular lesions and opting for wide resection in axial lesions. This principle is reflected in the results obtained as both our curettages performed on axial lesions recurred, while lesions submitted to wide resection tended to have lower recurrence, metastatic disease, and mortality rates.

However, the complication rates associated with wide resection procedures and complex reconstruction are higher when compared to curettage procedures. This is, therefore, the main attraction and point in favor of curettage surgery, the lower morbidity in patients who have long survivals regardless of the location of the disease [23-25].

On the other hand, we found some data that differed from the results usually described, namely regarding metastatic disease. From our population of 21 patients, we had four cases of metastatic disease associated with local recurrence; this corresponds to 19% of metastatic disease. This is atypically high compared to the reported rates of 0 to 16% [6,12,14]. Of these four cases of metastatic disease, two were located in the proximal femur and were treated with curettage, 1 was an acetabular lesion that also underwent curettage, and 1 was a dorsal vertebra with an R0 excision. The authors propose two explanations for these results: First, a small sample bias may account for this high rate of metastatic disease. Secondly, we may be looking at "false LG-CS"; it is known that these tumors are difficult to distinguish clinically, imaging, and histologically and that they may share characteristics with both benign and more malignant lesions [4,7]. These lesions may also already present or develop foci of upgraded chondrosarcoma. Thus, we may face entities of higher malignancy that we cannot initially distinguish. In all cases of metastatic disease, lesion upgrading was documented: two cases of G2, one case of G3, and one case of dedifferentiated chondrosarcoma. For this reason, we cannot advocate curettage in axial lesions since the risk of curettage of higher-grade chondrosarcoma is real and cannot be neglected; in these cases, higher recurrence rates may be expected.

## Conclusions

Our small series of 21 cases does not allow us to define the ideal treatment for this disease. However, we were able to highlight some findings that may contribute to guiding the path for future research in this pathology: a trend towards longer survival, less recurrence and metastatic disease after wide resection, more complications, and greater severity of these with wide resection. Nevertheless, a long overall survival can be expected regardless of the procedure or location. We also obtained a higher rate of metastatic disease than described in the literature; this, coupled with a misgrading rate of 9%, reflects the difficulty of preoperative diagnosis and the risk of treating high-grade chondrosarcomas as an LG-CS. When addressing relatively rare pathology subgroups, the available literature tends to be of low quality and retrospective. Multicenter clinical studies involving a larger number of patients with a longer follow-up are needed.
